# Hippocampal avoidance whole-brain radiotherapy with simultaneous integrated boost in lung cancer brain metastases and utility of the Hopkins verbal learning test for testing cognitive impairment in Chinese patients: a prospective phase II study

**DOI:** 10.1186/s12885-024-12559-1

**Published:** 2024-07-26

**Authors:** Zhuoran Li, Jianyang Wang, Lei Deng, Yirui Zhai, Tao Zhang, Nan Bi, Jingbo Wang, Xin Wang, Wenyang Liu, Zefen Xiao, Dongfu Chen, Jima Lv, Qinfu Feng, Wenqing Wang, Zongmei Zhou

**Affiliations:** Department of Radiation Oncology, National Cancer Center/National Clinical Research Center for Cancer/Cancer Hospital, Chinese Academy of Medical Sciences and Peking Union Medical College, No.17 Panjiayuan Nanli, Chaoyang District, Beijing, 100021 P.R. China

**Keywords:** Lung neoplasms, Brain metastasis, Simultaneous integrated boost, Hippocampal avoidance, Cognitive function

## Abstract

**Background:**

This study aimed to evaluate the efficiency of hippocampal avoidance whole-brain radiotherapy with a simultaneous integrated boost (HA-WBRT-SIB) treating brain metastases (BM) and utility of the Hopkins Verbal Learning Test-Revised (HVLT-R) (Chinese version) in Chinese lung cancer patients.

**Methods:**

Lung cancer patients with BM undergone HA-WBRT-SIB at our center were enrolled. Brain magnetic resonance imaging, The HVLT total learning score, and side effects were evaluated before radiotherapy and 1, 3, 6, and 12 months after radiotherapy. This study analyzed the overall survival rate, progression-free survival rate, and changes in HVLT-R immediate recall scores.

**Results:**

Forty patients were enrolled between Jan 2016 and Jan 2020. The median follow-up time was 14.2 months. The median survival, progression-free survival, and intracranial progression-free survival of all patients were 14.8 months, 6.7 months and 14.8 months, respectively. Multivariate analysis indicated that male sex and newly diagnosed stage IV disease were associated with poor overall survival and progression-free survival, respectively. HVLT-R scores at baseline and 1, 3, and 6 months after radiotherapy were 21.94 ± 2.99, 20.88 ± 3.12, 20.03 ± 3.14, and 19.78 ± 2.98, respectively. The HVLT-R scores at 6 months after radiotherapy decreased by approximately 9.8% compared with those at baseline. No grade 3 toxicities occurred in the entire cohort.

**Conclusions:**

HA-WBRT-SIB is of efficiency and cognitive-conserving in treating Chinese lung cancer BM.

**Trial registration:**

This study was retrospectively registered on ClinicalTrials.gov in 24th Feb, 2024. The ClinicalTrials.gov ID is NCT06289023.

## Background

Lung cancer is the second common malignancy and the leading cause of cancer-related deaths worldwide. [1]. Brain metastases (BM) originating from lung cancer have a high occurrence rate and are the primary cause of death. The BM occurrence rate ranges from 30 to 50% in lung cancer, with an average survival period of only 2–3 months without treatment. The median survival time extends to approximately 4–10 months after effective treatment [2].

Whole-brain radiotherapy (WBRT) is the standard palliative treatment by alleviating neurological symptoms and improving overall survival (OS) for BM. Compared with stereotactic radiosurgery (SRS), low intracranial local control and cognitive function impairment limits its application. However, only 3.3% of BM are located within 5 mm of the hippocampus [3]. Therefore, hippocampal avoidance (HA) radiotherapy might be considered when the tumor is located more than 1 cm from the hippocampus. Prospective clinical studies showed on HA prophylactic cranial irradiation (PCI) and HA-WBRT did not increase the rate of BM or decrease OS and could protect the cognitive function of patients [4–7].

Early clinical trials suggested that simultaneous integrated boost (SIB) radiotherapy at a dose of 50 Gy is safe, tolerable, and may have superior efficacy compared to WBRT [8]. A recent meta-analysis of small cell lung cancer indicated that the OS with SRS combined with WBRT was not significantly different from that with SRS alone [9]. As the intracranial recurrence rate after WBRT is significantly lower than that after SRS, this study aimed to assess the efficiency of HA-WBRT-SIB for BM in terms of minimizing cognitive impairment while maximizing intracranial control.

## Methods

### Patient eligibility

The inclusion Criteria were as follows: (1) pathologically diagnosed primary lung cancer with brain metastasis confirmed via magnetic resonance imaging (MRI); (2) 18–75 years old; (3) BM outside a 10 mm margin around either hippocampus; (4) at least one BM existed if prior resection of BM was done; (5) BM measuring less than 5.0 cm in maximal extent; and (6) Eastern Cooperative Oncology Group (ECOG) performance status scores of 0–2. The exclusion Criteria were as follows: (1) previous brain radiotherapy or brain metastasis resection; (2) history of malignancies other than lung cancer; (3) radiographic evidence of hydrocephalus or other architectural distortion of the ventricular system, leptomeningeal metastases, and (4) presence of other serious illnesses such as acute myocardial infarction, severe arrhythmia, or psychiatric disorders within the past 6 months.

All patients underwent pre-radiotherapy examinations, including complete blood count, liver and kidney function tests, tumor markers related to lung cancer, chest-abdomen-pelvis computed tomography (CT), and contrast-enhanced brain MRI. This study was approved by our Institutional Review Board (Approval Number: 17/094-7845. Board Name: National GCP Center for Anticancer Drugs, The Independent Ethics Committee. Board Affiliation: National Cancer Center/Cancer Hospital, Chinese Academy of Medical Sciences and Peking Union Medical College). Informed consent to participate was obtained from all of the participants in the study.

### Treatment

All patients were immobilized in the supine position using customized devices. CT and MRI scans with a 2-mm slice thickness were routinely performed, and CT and MRI images were fused using the Phillips Pinnacle system. Tumor gross target volume (GTV) was delineated using T1-enhanced and T2-FLAIR images. A 3 mm three-dimensional expansion formed the boost area. The clinical target volume (CTV) was defined as the whole-brain parenchyma, and the planning target volume was delineated using the CTV plus a 3-mm margin in all directions, excluding the HA regions. The hippocampus was delineated based on the RTOG 0933 guidelines using T1-BRAVO-weighted sequences [5]. For definition of HA region, we expanded the bilateral hippocampus by 2 mm in three-dimension to generate planning risk volume (PRV) of hippocampus, in order to ensure sufficient dose reduction space and compensation for positioning errors. Patients underwent irradiation using image-guided radiotherapy, receiving a total dose of 30–36 Gy delivered in 18–20 fractions to the whole brain (CTV) [10–12], while the dose to the GTV were boosted to 44–52 Gy in 18–20 fractions, five times a week. In preliminary cohort of 5 patients, we constrained the optimal mean dose (Dmean) to the bilateral hippocampus below or equal to 8 Gy and maximal dose (Dmax) to the hippocampus not exceeding 10 Gy, Dmean of bilateral hippocampus PRV ≤ 9 Gy and Dmax ≤ 12 Gy. However, the parameters of 4 patients plan did not meet this dose limitation requirement. Then, we set up criteria for Dmean to bilateral hippocampus and PRV optimally ≤ 10 Gy and ≤ 12 Gy respectively, and mandatory Dmax to bilateral hippocampus and PRV ≤ 14 Gy and ≤ 20 Gy respectively. Dose constraints for other organs at risk are outlined as following: the maximum dose to the lens should be < 9 Gy; the maximun dose to the spinal cord should be < 40 Gy; the maximun dose to the brain stem should be < 50 Gy and the maximun dose to the optic nerve pathways should be < 50 Gy. Treatment delivery that exceeds defined limits will constitute a major protocol violation, which need approval of more than 2 radiation oncologist and primary investigator before the dose was delivered. MU was not limited to the optimization process of the VMAT program.

Systemic treatments were administered according to the guidelines based on the pathological type, immunohistochemical results, and molecular mutation testing.

### Endpoints

The primary endpoint was OS, which was defined as the time from the end of brain radiotherapy to death from any cause. The secondary endpoints were progression-free survival (PFS), median intracranial progression-free survival (iPFS), and HVLT-R total learning score. PFS was defined as the time from the end of brain radiotherapy to tumor progression or death from any cause, and iPFS was defined as the time from the end of brain radiotherapy to intracranial tumor progression or death from any cause. Pretreatment evaluations, including blood tests, chest CT, abdominal ultrasound, and brain MRI, were conducted 2 weeks before treatment. Post-treatment MRI was performed at 1, 3, 6, and 12 months. The brain metastatic lesion response was assessed according to the Response Evaluation Criteria in Solid Tumors 1.1.

For the cognitive function evaluation, Hopkins Verbal Learning Test-Revised (HVLT-R) (Chinese version) were used to testy its utility in Chinese patients [13]. The HVLT-R immediate recall scores were obtained at baseline and 1, 3, and 6 months after treatment [14]. The HVLT-R consists of a 12-item word list, composed of four words from each of the three semantic categories. The word list is read to the subject at the approximate rate of one word every 2 s. The patient’s free recall of the list is recorded and each correct word scores 1 point (immediate recall scores, trial 1). The same procedure is repeated for two more trials (trial 2 and 3). The HVLT scores were calculated as the sum of trials 1, 2, and 3. Adverse events were assessed using the Common Terminology Criteria for Adverse Events 5.0.

### Data analysis

Continuous variables are presented as means and standard deviations, while categorical variables are expressed as frequency and composition ratios or rates. Survival analysis was conducted using the Kaplan–Meier method. Independent prognostic factors for OS and PFS were identified using Cox stepwise regression analysis with 95% confidence interval (95%CI) for variables with a P-value < 0.25 in univariate Cox analyses. All data analyses were performed using SPSS (v25.0; IBM, Armonk, NY, USA) and R (v4.0.4; R Foundation, Vienna, Austria). P-value < 0.05 considered statistically significant.

## Results

### Patient baseline

Forty eligible patients were enrolled between Jan 2016 and Jan 2020. Table 1 shows the baseline patient characteristics. Most patients (70%) were male, with an average age of 69.95 years, and the majority (90%) had an ECOG performance score of 1. Of all the patients, 25 had non-small cell lung cancer (NSCLC) and 10 had epidermal growth factor receptor (EGFR) or Anaplastic Lymphoma Kinase (ALK) mutations. At the initial diagnosis, 17 patients (42.5%) had distant metastases and 18 (45%) had bilateral brain metastases before brain radiotherapy.

**Table 1:** Patient baseline characteristics

Characteristic (*N* = 40)	
Age (years, Mean ± SD)	60.95 ± 10.66
Gender	
Male	28 (70.0)
Female	12 (30.0)
ECOG	
1	36 (90.0)
2	4 (10.0)
Smoking History	26 (65.0)
Alcohol History	17 (42.5)
Pathological Type	
Small Cell Lung Cancer	15 (37.5)
Non-Small Cell Lung Cancer	25 (62.5)
Molecular Mutation	
None	24 (60.0)
EGFR	8 (20.0)
ALK	2 (5.0)
Unknown	6 (15.0)
Number of Brain Metastases	
1	16 (40.0)
2–4	16 (40.0)
5–10	8 (20.0)
Size of Brain Metastases (cm, Mean ± SD)	1.50 ± 0.91
Location of Brain Metastases	
Unilateral	22 (55.0)
Bilateral	18 (45.0)
Initial Staging	
I	3 (7.5)
II	1 (2.5)
III	19 (47.5)
IV	17 (42.5)

*Abbreviation* ECOG, Eastern Cooperative Oncology Group; EGFR, Epidermal Growth Factor Receptor; ALK Anaplastic Lymphoma Kinase

### Treatment overview

Table 2 summarizes the treatment details. Hippocampal doses (Dmax and Dmean) were 12.24 ± 2.06 Gy and 8.92 ± 1.79 Gy. Only one patient did not receive systemic treatment prior to brain radiotherapy. Volumetric modulated arc therapy (VMAT) was used in 17 cases (42.5%) and helical tomotherapy (TOMO) in 23 (57.5%). Doses for the boost area and whole-brain were 52.23 ± 6.52 Gy and 32.48 ± 2.67 Gy, respectively. The most common prescription was 32.4 Gy/1.8 Gy/18f for whole-brain and 50.4 Gy/2.8 Gy/18f for boost.

**Table 2:** Treatment details

Parameters (*N* = 40)	
Pre-radiotherapy Anti-tumor Treatment	
Thoracic Surgery	10 (25.0)
Thoracic Radiotherapy	18 (45.0)
Systemic Therapy	39 (97.5)
Radiotherapy Technique	
TOMO	23 (57.5)
VMAT	17 (42.5)
Boost Area Dose (EQD_10/2_, Gy)	
Mean ± SD	52.23 ± 6.52
Whole Brain Dose (EQD_10/2_, Gy)	
Mean ± SD	32.48 ± 2.67
Hippocampal Dose (Gy)	
Dmax ± SD	12.24 ± 2.06
Dmean ± SD	8.92 ± 1.79
Hippocampal PRV Dose (Gy)	
Dmax ± SD	16.81 ± 3.45
Dmean ± SD	10.02 ± 2.08
Intracranial Best Treatment Response	
CR	7 (17.5)
PR	16 (40.0)
SD	8 (20.0)
PD	4 (10.0)
Not Evaluated	5 (12.5)
First Site of Brain Radiotherapy Failure	
Lung	15 (37.5)
Brain	11 (27.5)
Bone	7 (17.5)
Liver	7 (17.5)
Other	6 (15.0)

*Abbreviation* VMAT, Volumetric Modulated Arc Therapy; TOMO, Helical Tomotherapy; EQD_10/2_, Equivalent Dose in 2 Gy Fractions, α/β = 10, equivalent dose when delivered in 2 Gy fractions; CR, Complete Response; PR, Partial Response; SD, Stable Disease; PD, Progressive Disease

### Survival outcomes

**Fig. 1:**
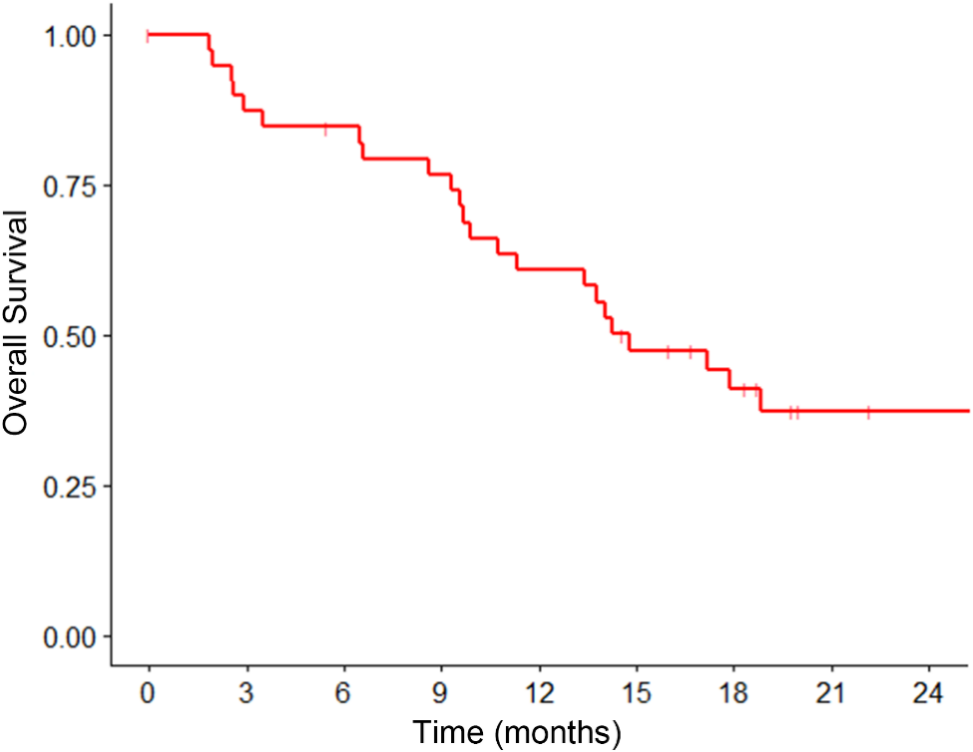
Overall survival of enrolled patients

**Fig. 2:**
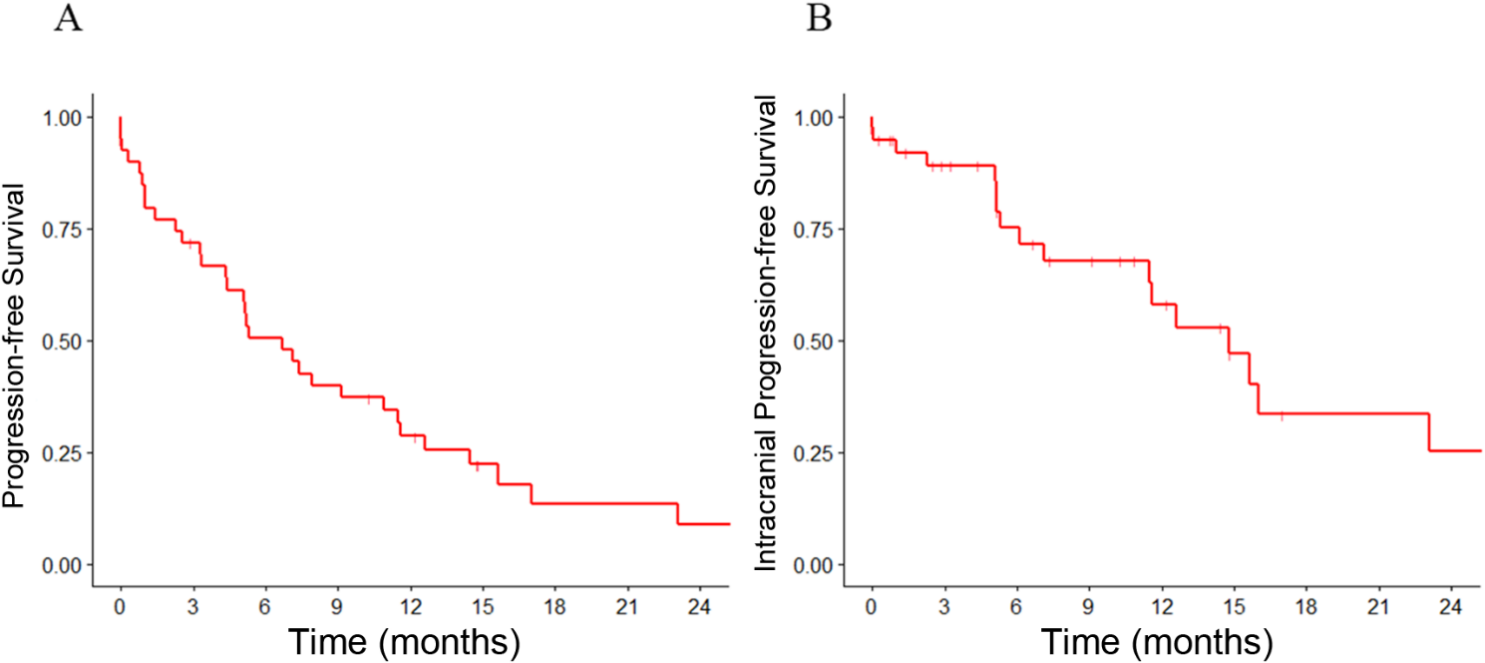
Progression-free survival (PFS) (**A**) and intracranial progression-free survival (iPFS) (**B**) for the enrolled patients

The median follow-up was 14.2 months. The median OS was 14.8 months with a 1-year OS of 60.8% (Fig. 1). Median PFS and iPFS were 6.7 and 14.8 months, with 1-year PFS and iPFS at 28.7% and 58.1%, respectively (Fig. 2). Seventeen patients (five SCLC, twelve NSCLC) experienced intracranial progression: 10 in the boost area, 12 outside the boost area, and five in both areas. No recurrences were observed in the HA area. Extracranial progression occurred in 26 patients, with the common sites being the lungs (16 patients), bones (eight patients), and liver (seven patients). In univariate analysis (Table 3), sex was associated with OS (HR 4.70, 95% CI 1.38–15.98) and tumor stage correlated with PFS (HR 2.27, 95% CI 1.08–4.78). In multivariate analysis, male sex (HR 4.09, 95% CI 1.16–14.41) and stage IV at diagnosis (HR 2.32, 95% CI 1.09–4.91) remained associated with poorer OS and PFS, respectively.

**Table 3:** Univariate and multivariate analyses of overall survival and progression-free survival

Variable	Univariate Analysis		Multivariate Analysis	
	HR (95% CI)	*P*-value	HR (95% CI)	*P*-value
**Overall Survival**				
Age, > 60 vs. ≤ 60	1.64 (0.71–3.80)	0.24	1.01 (0.97–1.06)	0.53
Gender, Male vs. Female	4.70 (1.38–15.98)	0.007*	4.09 (1.16–14.41)	0.03*
ECOG Performance Status, 2 vs. 1	2.38 (0.79–7.11)	0.16	1.76 (0.58–5.36)	0.32
Pathology, Small Cell vs. Non-Small Cell	0.89 (0.38–2.06)	0.78		
Stage, IV vs. I-III	1.94 (0.85–4.44)	0.12	2.00 (0.86–4.63)	0.11
Molecular Mutation, Present vs. Absent/Unknown	1.03 (0.41–2.62)	0.95		
Number of Brain Metastases, Multiple vs. Single	1.34 (0.58–3.11)	0.49		
**Progression-Free Survival**				
Age, > 60 vs. ≤ 60	0.969 (0.47–1.98)	0.93		
Gender, Male vs. Female	1.75 (0.80–3.82)	0.15	1.78 (0.81–3.92)	0.15
ECOG Performance Status, 2 vs. 1	1.53 (0.46–5.12)	0.52		
Pathology, Small Cell vs. Non-Small Cell	1.08 (0.53–2.22)	0.84		
Stage, IV vs. I-III	2.27 (1.08–4.78)	0.03*	2.32 (1.09–4.91)	0.03*
Molecular Mutation, Present vs. Absent/Unknown	1.20 (0.53–2.68)	0.67		
Number of Brain Metastases, Multiple vs. Single	0.71 (0.34–1.45)	0.35		

^*^Statistically significant (*p* < 0.05)

*Abbreviation* ECOG, Eastern cooperative oncology group

### Toxicity and cognitive function

**Fig. 3:**
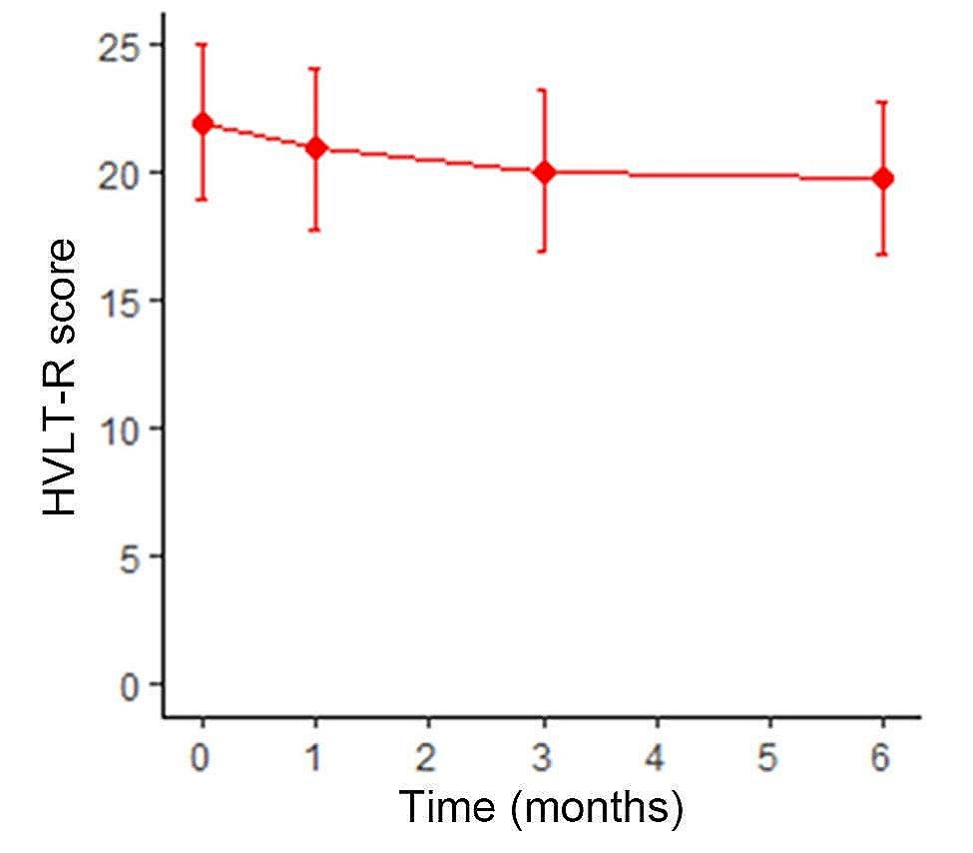
Hopkins Verbal Learning Test-Revised (HVLT-R) scores for the enrolled patients

No grade 3 or above toxicities were observed. Common adverse events included nausea (27.5%), dizziness (25.0%), headaches (17.5%), and hair loss (12.5%). Seven patients experienced memory impairment, with one case of grade 2 impairment, possibly related to advanced age and a larger boost area. Of the 32 patients who survived for > 6 months, 28 had complete immediate recall of the HVLT-R scores. The mean baseline HVLT-R score was 21.94 ± 2.99. Scores at 1-, 3- and 6-month post-radiotherapy were 20.88 ± 3.12, 20.03 ± 3.14, and 19.78 ± 2.98, respectively. At 6 months, the HVLT-R score declined by approximately 9.8% compared to that at baseline (Fig. 3).

## Discussion

WBRT is the standard treatment for brain metastases originating from lung cancer. However, an inadequate dose for metastatic lesions during WBRT results in intracranial progression in approximately 50% of patients within 6 months [15]. Additionally, compared to SRS, WBRT is associated with increased cognitive function impairment, primarily related to hippocampal exposure [16]. Therefore, a boost to metastatic lesions based on WBRT, along with HA, when lesions are distant from the hippocampus, has become a potential radiotherapy technique to improve local control and reduce side effects. In this study, with metastatic lesions at least 1 cm away from the hippocampus and radiotherapy meeting the dose requirements for HA, the median OS and PFS were 14.8 months and 6.7 months, respectively, which were superior to historical controls treated with systemic chemotherapy alone [17]. The median iPFS was 14.8 months, similar to that of patients with EGFR mutations receiving targeted therapy [15].

Upon recognizing the close relationship between the hippocampus and cognitive function, HA research was initially conducted in patients undergoing WBRT. The RTOG 0933 trial included patients with malignant brain metastases who underwent WBRT with HA at 30 Gy. This study found that the degree of decline in HVLT-R scores for patients in the HA group was significantly lower than that in historical controls without sacrificing OS, confirming the significance of HA in cognitive function preservation [5]. However, three patients in the RTOG 0933 study experienced progression in the HA area, which was higher than that in our study. This difference may be attributed to the RTOG 0933 trial using a larger HA area, looser brain metastasis position restrictions, and the absence of SIB. In our study, the HVLT-R score decreased by 9.8% at 6 months compared to that at baseline, which was higher than the 3.0% decrease in the RTOG 0933 trial. The primary reason for this might be the unavoidable increase in the hippocampal dose in our study owing to the addition of a boost to WBRT. However, the decline in HVLT-R scores remained significantly lower than that in the historical controls at 30–50% [18]. Subsequent results from the NRG CC001 study further confirmed that HA in WBRT, combined with memantine, could further reduce the risk of cognitive impairment [6]. For PCI in NSCLC, two phase III trials showed contradictory results. The PREMER study indicated that HA in PCI did not reduce the intracranial failure rate or OS and could protect the cognitive function of patients in the long term, whereas another study indicated that HA did not show advantages in cognitive function protection [4, 7]. Further studies are required to elucidate the role of HA in PCI.

The HVLT-R score has been widely used in the aforementioned studies to assess changes in cognitive function in patients receiving brain radiotherapy. The application of the HVLT-R and evaluation of cognitive impairment have also been validated in the Chinese population [13]. However, no previous studies have evaluated its effectiveness in Chinese cancer patients. The current study demonstrated that the HVLT-R can be used to evaluate the impact of radiotherapy on cognitive function in Chinese lung cancer patients with brain metastases. Both our study and the aforementioned studies suggest that minimizing the hippocampal dose consistently benefits patients in terms of their cognitive function.

Owing to the significant impairment of cognitive function caused by WBRT and insufficient local doses, SRS has gained attention for the treatment of brain metastases. An early phase III study from Japan found that when there were no more than four brain metastases, SRS combined with WBRT reduced the 1-year intracranial control rate from 76.4 to 46.8% but did not improve OS compared with SRS alone [19]. Subsequent studies have yielded similar conclusions—for patients with limited brain metastases, SRS combined with WBRT improved local control but did not enhance OS, with cognitive function impairment as a drawback [20–24]. Therefore, the 2022 ASTRO guidelines do not routinely recommend WBRT for patients with limited brain metastases who have undergone SRS. However, studies have shown that even for lung cancer patients with brain metastases who have a favorable prognosis, SRS combined with WBRT can increase the median survival time, and the benefits of WBRT should not be overlooked [25]. The ASTRO guidelines also emphasize that SRS combined with WBRT can be considered to maximize intracranial control.

With the advent of advanced radiotherapy techniques such as VMAT and TOMO, delivering an SIB to metastatic lesions based on WBRT, along with HA, has become feasible [26, 27]. SIB-HA not only increased the tumor dose to reduce local failure but also minimized the hippocampal dose to protect cognitive function. Our results demonstrated the feasibility of HA when the tumor lesions were > 1 cm away from the hippocampal region, and the risk of hippocampal failure was low. During the follow-up period, none of the 40 patients experienced progression in the HA area. The intracranial control rate was 77.5%, with both median iPFS and OS of 14.8 months, demonstrating the preliminary effectiveness and safety of this treatment strategy. Cox analysis revealed that only sex and initial tumor stage were associated with OS and PFS, respectively, whereas other factors showed no significant associations. A retrospective analysis by Lebow et al. indicated that using a similar strategy to treat brain metastases (mostly NSCLC) resulted in a median iPFS and OS of 11.4 months and 19.6 months, respectively. Cognitive function impairment occurred in 15.6% of patients during follow-up, with one case of grade 3, potentially owing to some patients receiving previous brain radiotherapy [28]. In another retrospective study, treatment outcomes of SIB-HA and WBRT were ompared, revealing that the former extended median iPFS and OS from 6.4 months and 6.2 months to 13.5 months and 9.9 months, respectively. However, 6.5% of the patients experienced metastasis within the HA area [29]. The research group planned to conduct a prospective Phase II HIPPORAD study to evaluate whether this strategy could reduce cognitive functional impairment. The study planned to enroll patients with ≥ 4 brain metastatic lesions, assigning them in a 1:1 ratio to the SIB ± HA group. The results of this study are expected to provide additional evidence for SIB-HA [30]. Recently published results from a single-arm phase II study of SIB-HA for brain metastases showed that despite a mild decrease in HVLT-R score 3 months after treatment, the PFS and OS for the entire group were only 2.9 months and 9 months, respectively. The short survival time resulted in a low completion rate of cognitive function assessments in this study, and further validation is warranted for these conclusions [31].

Our study had some limitations. First, this prospective phase II study had a small sample size and a long enrollment span, inevitably leading to selection bias and inconsistent treatment choices among the patients. However, our study exclusively included patients with brain metastases originating from lung cancer, which reduced the heterogeneity when compared to retrospective studies involving brain metastases from different cancers. Second, this study had a single-arm design without selecting patients who did not receive HA as a control. However, multiple studies on HA have proven its effectiveness in reducing cognitive impairment. Therefore, we believe that SIB-HA can provide cognitive benefits without reducing treatment efficacy. Third, the constraints for hippocampus were hard to achieve and most patients in this study didn’t meet the requirements. A constraint of Dmax < 20 Gy and Dmean < 12 Gy for hippocampus PRV might be more appropriate for HA-WBRT-SIB.

## Conclusions

In summary, SIB-HA is a safe and effective treatment method for lung cancer brain metastases that can potentially reduce the impact of radiotherapy on cognitive function. Given the diversity of treatment modalities for lung cancer brain metastases and the feasibility of our study, the results require further validation in larger prospective studies.

## Data Availability

Research data are stored in an institutional repository and will be shared upon reasonable request to the corresponding author.

## Abbreviations

BM: Brain metastases
WBRT: Whole-brain radiotherapy
OS: Overall survival
SRS: Stereotactic radiosurgery
HA: Hippocampal avoidance
PCI: Prophylactic cranial irradiation
SIB: Simultaneous integrated boost
HVLT: R-Hopkins Verbal Learning Test-Revised
MRI: Magnetic resonance imaging
ECOG: Eastern Cooperative Oncology Group
CT: Computed tomography
GTV: Gross target volume
CTV: Clinical target volume
PRV: Planning risk volume
Dmean: Mean dose
Dmax: Maximum dose
PFS: Progression-free survival
iPFS: Intracranial progression-free survival
NSCLC: Non-small cell lung cancer
EGFR: Epidermal growth factor receptor
ALK: Anaplastic Lymphoma Kinase
VMAT: Volumetric modulated arc therapy
TOMO: Helical tomotherapy
